# Time dependent outcomes modeling in a real-world analysis of the molecular tumor board at University Cancer Center Hamburg (2016-2022)

**DOI:** 10.1093/oncolo/oyag078

**Published:** 2026-03-31

**Authors:** Janna-Lisa Velthaus-Rusik, Jan Frederic Weller, Melisa Hoshaber, Benjamin Schmidt, Corinna Albers-Leischner, Malte Kriegs, Sonja Loges, Stefan Bartels, Catarina Schlüter, Ronald Simon, Alexander E Volk, Christian Kubisch, Frank Ückert, Layla Riemann, Katja Weisel, Gunhild von Amsberg, Winfried Alsdorf, Carsten Bokemeyer, Maximilian Christopeit

**Affiliations:** Department of Medicine II (Oncology, Hematology, Bone Marrow Transplantation with Section Pneumology), University Medical Center Eppendorf, Hamburg, 20246, Germany; University Cancer Center (UCC) Hamburg, University Medical Center Eppendorf, Hamburg, 20246, Germany; Department of Medicine II (Oncology, Hematology, Bone Marrow Transplantation with Section Pneumology), University Medical Center Eppendorf, Hamburg, 20246, Germany; University Cancer Center (UCC) Hamburg, University Medical Center Eppendorf, Hamburg, 20246, Germany; Department of Medicine II (Oncology, Hematology, Bone Marrow Transplantation with Section Pneumology), University Medical Center Eppendorf, Hamburg, 20246, Germany; University Cancer Center (UCC) Hamburg, University Medical Center Eppendorf, Hamburg, 20246, Germany; Department of Medicine II (Oncology, Hematology, Bone Marrow Transplantation with Section Pneumology), University Medical Center Eppendorf, Hamburg, 20246, Germany; University Cancer Center (UCC) Hamburg, University Medical Center Eppendorf, Hamburg, 20246, Germany; Department of Medicine II (Oncology, Hematology, Bone Marrow Transplantation with Section Pneumology), University Medical Center Eppendorf, Hamburg, 20246, Germany; University Cancer Center (UCC) Hamburg, University Medical Center Eppendorf, Hamburg, 20246, Germany; University Cancer Center (UCC) Hamburg, University Medical Center Eppendorf, Hamburg, 20246, Germany; Department of Radiotherapy, University Medical Center Eppendorf, Hamburg, 20246, Germany; DKFZ-Hector Cancer Institute at the University Medical Center Mannheim, Mannheim, 68167, Germany; Department of Personalized Medical Oncology (A420), German Cancer Research Center (DKFZ), German Center for Lung Research (DZL), Heidelberg, 69120, Germany; University Hospital Mannheim, Medical Faculty Mannheim, Mannheim, 68167, Germany; University Cancer Center (UCC) Hamburg, University Medical Center Eppendorf, Hamburg, 20246, Germany; Local Cancer Registry (KKR), University Medical Center Eppendorf, Hamburg, 20246, Germany; University Cancer Center (UCC) Hamburg, University Medical Center Eppendorf, Hamburg, 20246, Germany; Local Cancer Registry (KKR), University Medical Center Eppendorf, Hamburg, 20246, Germany; University Cancer Center (UCC) Hamburg, University Medical Center Eppendorf, Hamburg, 20246, Germany; Department of Pathology, University Medical Center Eppendorf, Hamburg, 20246, Germany; University Cancer Center (UCC) Hamburg, University Medical Center Eppendorf, Hamburg, 20246, Germany; Department of Human Genetics, University Medical Center Eppendorf, Hamburg, 20246, Germany; University Cancer Center (UCC) Hamburg, University Medical Center Eppendorf, Hamburg, 20246, Germany; Department of Human Genetics, University Medical Center Eppendorf, Hamburg, 20246, Germany; University Cancer Center (UCC) Hamburg, University Medical Center Eppendorf, Hamburg, 20246, Germany; Department of Applied Medical Informatics, University Medical Center Eppendorf, Hamburg, 20246, Germany; University Cancer Center (UCC) Hamburg, University Medical Center Eppendorf, Hamburg, 20246, Germany; Department of Applied Medical Informatics, University Medical Center Eppendorf, Hamburg, 20246, Germany; Department of Medicine II (Oncology, Hematology, Bone Marrow Transplantation with Section Pneumology), University Medical Center Eppendorf, Hamburg, 20246, Germany; University Cancer Center (UCC) Hamburg, University Medical Center Eppendorf, Hamburg, 20246, Germany; Department of Medicine II (Oncology, Hematology, Bone Marrow Transplantation with Section Pneumology), University Medical Center Eppendorf, Hamburg, 20246, Germany; University Cancer Center (UCC) Hamburg, University Medical Center Eppendorf, Hamburg, 20246, Germany; Department of Medicine II (Oncology, Hematology, Bone Marrow Transplantation with Section Pneumology), University Medical Center Eppendorf, Hamburg, 20246, Germany; University Cancer Center (UCC) Hamburg, University Medical Center Eppendorf, Hamburg, 20246, Germany; Department of Medicine II (Oncology, Hematology, Bone Marrow Transplantation with Section Pneumology), University Medical Center Eppendorf, Hamburg, 20246, Germany; University Cancer Center (UCC) Hamburg, University Medical Center Eppendorf, Hamburg, 20246, Germany; Department of Medicine II (Oncology, Hematology, Bone Marrow Transplantation with Section Pneumology), University Medical Center Eppendorf, Hamburg, 20246, Germany; University Cancer Center (UCC) Hamburg, University Medical Center Eppendorf, Hamburg, 20246, Germany

**Keywords:** molecular tumor board, precision oncology, predictive oncology, time-dependent covariate, immortal time bias, Mantel–Byar, Simon–Makuch

## Abstract

**Introduction:**

Molecular tumor boards (MTBs) integrate clinical, pathological, and molecular data to prioritize therapy options. Because MTB-guided treatment starts at varying time points after MTB discussion, vulnerabilities to immortal time bias occur if treatment delay is ignored.

**Methods:**

Retrospective survival analysis of 949 consecutive individuals discussed at the University Cancer Center Hamburg MTB (2016-2022). Index date (t_0_) was the first MTB discussion. Therapy initiation was modeled as a time-dependent exposure using Mantel-Byar methods and visualized with Simon–Makuch plots. Therapy strata were priority 1 targeted, priority 2 targeted, and non-targeted therapy.

**Results:**

Among solid tumors (*n* = 854), targeted therapy was implemented in 24% and was off-label in 51%. ORR/DCR were 30.8%/61% for priority 1, 11.4%/36.4% for priority 2, and 37.5%/52.1% for non-targeted therapy (*P* = .017). The progression-free survival ratio (MTB-directed therapy vs immediately preceding line) exceeded 1.3 in 63% of targeted therapy recipients. From the MTB date, median survival_MTB_ was 16.5 months (priority 1), 15.5 months (priority 2), and 11.0 months (non-targeted). In Mantel-Byar analysis, progression-free survival_SimonMakuch_ favored priority 1 (*P* = .002) with 1-year rates of 28%, 12%, and 15% for priority 1, priority 2, and non-targeted therapy, respectively. One-year overall survival_SimonMakuch_ was 52%, 34%, and 42%. In a time-dependent Cox model, priority 1 was associated with lower risk of death versus no targeted post-MTB therapy (hazard ratio 0.80, 95% confidence interval 0.64-0.99, *P* = .038).

**Conclusions:**

After correction for immortal time bias, implementation of the top-priority MTB recommendation was associated with improved disease control and survival signals, although confounding and selection effects remain key limitations.

Implications for Practice

- Accurate outcomes estimation of MTB-guided therapy requires controlling for immortal time bias when treatment starts after MTB consultation.

## Introduction

Personalized cancer care increasingly relies on tumor molecular profiling to inform targeted treatment strategies. While the relative utility of single-gene assays, targeted panels, and broader approaches such as exome or genome sequencing is still being evaluated, molecular aberrations have enabled entity-agnostic or oligo-indicative approvals, including immune checkpoint inhibition for tumors with defective mismatch repair or high tumor mutational burden and kinase inhibition for defined oncogenic fusions (eg, NTRK) or mutations (eg, BRAFV600E).^1–6^ In addition, complementary approaches such as proteomic/kinomic/epigenetic profiling and expanded immunohistochemistry (eg, for monoclonal antibodies and antibody-drug conjugates) are increasingly explored or integrated into clinical decision-making around MTBs.^7–17^

In Germany, precision oncology is largely operationalized through molecular tumor boards (MTBs).^18^ Many MTBs are embedded in Centers for Personalized Medicine—Oncology and follow harmonized standard operating procedures developed within the German Network for Personalized Medicine (Deutsches Netzwerk für Personalisierte Medizin, DNPM).^19^^,^^20^ The nationwide establishment of precision oncology at university cancer centers has recently been supported by a state-governed model project within the statutory health insurances to enable broader access to tumor genome sequencing techniques.^21^ This ecosystem has fostered coordinated methodological development, ranging from comparisons of sequencing strategies to the integration of medical informatics for secure storage and analysis of molecular and clinical data.^22–24^

Conceptually, DNPM builds on earlier German initiatives such as MASTER (DKFZ), which introduced structured evidence grading (“Levels of Evidence”) to annotate MTB recommendations.^25^ For outcome assessment in heterogeneous real-world cohorts, the progression-free survival ratio (“PFS-Ratio”) was proposed as a within-person metric to approximate clinical benefit across individualized treatment trajectories.^26^ At the program level, the National Network for Genomic Medicine—Lung (nNGM) was among the first disease-specific initiatives to report improved overall survival within a structured precision oncology framework.^27^^,^^28^ Across published MTB cohorts, implementation rates and clinical outcomes vary considerably; a recent meta-analysis reported a pooled implementation rate of 20.8%, median overall survival of 13.5 months, and median progression-free survival of 4.5 months, with objective response rates ranging widely between studies.^29^^,^^30^ These heterogeneous data underline both the potential of MTB-guided care and the need for rigorous methodology in real-world outcome analyses.^31^^,^^32^

A key methodological challenge in MTB outcomes research is that MTB-guided therapies typically start after MTB discussion. If exposure groups are defined by a post-baseline event (therapy initiation), conventional Kaplan–Meier analyses are prone to immortal person-time bias and may overestimate treatment benefit. Mantel–Byar methods address this problem by treating therapy initiation as a time-dependent exposure, allocating person-time before therapy start to the unexposed risk set and switching individuals to the exposed stratum at treatment start; Simon–Makuch plots provide an appropriate visualization of these time-dependent comparisons.^33^^,^^34^ To strengthen causal interpretability in a real-world setting and to provide a reference analysis within the German MTB framework, we report outcomes for consecutive patients discussed in the molecular tumor board at the University Cancer Center (UCC) Hamburg between 2016 and 2022, with a particular emphasis on time-dependent modeling to reduce immortal time bias.

## Methods

### Study design and population

We conducted a retrospective cohort study of consecutive individuals discussed in the MTB of the UCC Hamburg, University Medical Center Hamburg-Eppendorf, between 2016 and 2022. During this period, 1074 MTB case discussions corresponded to 949 unique individuals. For the main analyses, we focused on individuals with solid tumors (*n* = 854). Individuals were followed from the MTB index date until death or last documented follow-up.

### Definitions and endpoints

Objective response rate (ORR) and disease control rate (DCR) were defined using standard response categories (complete response, partial response, stable disease, progressive disease) as documented in routine clinical care. Overall survival (OS_MTB_) was defined as time from the MTB index date to death from any cause, censoring individuals alive at last follow-up. Progression-free survival from MTB (PFS_MTB_) was defined as time from the MTB index date to documented progression or death, whichever occurred first.

For within-person assessment of benefit, we captured progression-free survival on the immediately preceding systemic therapy line (PFS_prior_) and progression-free survival on the MTB-directed implemented therapy line (PFS_MTB_), where available. The progression-free survival ratio was calculated as PFS_ratio_ = PFS_MTB_/PFS_prior_ as a pragmatic within-person metric; a threshold > 1.3 has been proposed to indicate clinically meaningful benefit in this setting.

### Index date and time-dependent exposure modeling

The index date (t_0_) was defined as the date of the first MTB discussion for each individual. The “implementation date of recommendation” refers to the therapy start date (initiation of the systemic treatment selected after MTB discussion). In time-dependent analyses, individuals contributed unexposed person-time from t_0_ until therapy start; from therapy start onward, they contributed exposed person-time to the corresponding therapy stratum (priority 1 targeted therapy, priority 2 targeted therapy, or non-targeted systemic therapy).

The interval between availability of the molecular sequencing report and MTB discussion occurred before t_0_ and was therefore not included in time-at-risk for time-to-event analyses. Sequencing results and pathology reports were treated as baseline information available at the MTB discussion.

### MTB recommendation process and prioritization

MTB recommendations were generated by multidisciplinary review of clinical history, tumor histopathology, and molecular findings (including mutations, copy number changes, and gene fusions, where assessed). Recommendations were annotated with evidence levels according to published frameworks. Priority 1 reflected the option judged most appropriate based on evidence level, expected on-target relevance for the tumor type, regulatory status and accessibility, anticipated toxicity in the individual clinical context, and feasibility. Priority 2 reflected the next best alternative when the top-ranked option could not be pursued or was expected to be less suitable.

### Therapy groups and clarification of “no therapy”

For descriptive and conventional (non-time-dependent) comparisons, individuals were categorized according to post-MTB systemic therapy documentation into (i) priority 1 targeted therapy implemented, (ii) priority 2 targeted therapy implemented, (iii) non-targeted systemic therapy implemented, or (iv) no post-MTB systemic therapy documented (“no therapy”). The “no therapy” category does not imply absence of actionable alterations alone; it may include individuals without actionable findings and individuals who did not start therapy for other clinical or logistical reasons (eg, clinical deterioration, competing treatment decisions, access constraints, or patient preference).

### Data sources, data capture, and quality control

MTB case information was maintained in a dedicated institutional MTB registry (structured data capture of case discussions and recommendations). Treatment exposure (therapy start), response assessments, progression dates, and survival status were abstracted from the electronic health record and, where available, complemented by the local cancer registry. Data curation included normalization of synonymous variant entries, plausibility checks for dates and intervals, and exclusion of non-informative values. Key timestamps (MTB date, therapy start date, progression date, death date, and last contact) were reconciled across data sources where possible. Data extraction and curation were performed by trained personnel; quality assurance consisted of consistency checks and targeted review of discrepant or missing key timestamps. Outcome and survival information were updated based on the most recent documentation available at the time of database lock for analysis.

### Statistical analysis

All analyses were performed in R (version 4.3.1). Descriptive statistics summarized tumor entities and molecular alterations. Group comparisons used Kruskal–Wallis tests for continuous variables and chi-squared tests for categorical variables; multiple testing was addressed using Benjamini–Hochberg correction (*q*-values). Time-to-event outcomes were estimated (i) using conventional Kaplan–Meier methods grouped by post-MTB therapy category and (ii) using Mantel-Byar time-dependent analyses, treating therapy initiation as a time-dependent exposure. Time-dependent comparisons were visualized using Simon–Makuch plots. Time-dependent Cox proportional hazards models were used to estimate hazard ratios with the therapy group as a time-dependent exposure.

## Results

### Cohort and clinical characteristics

**Figure 1.**
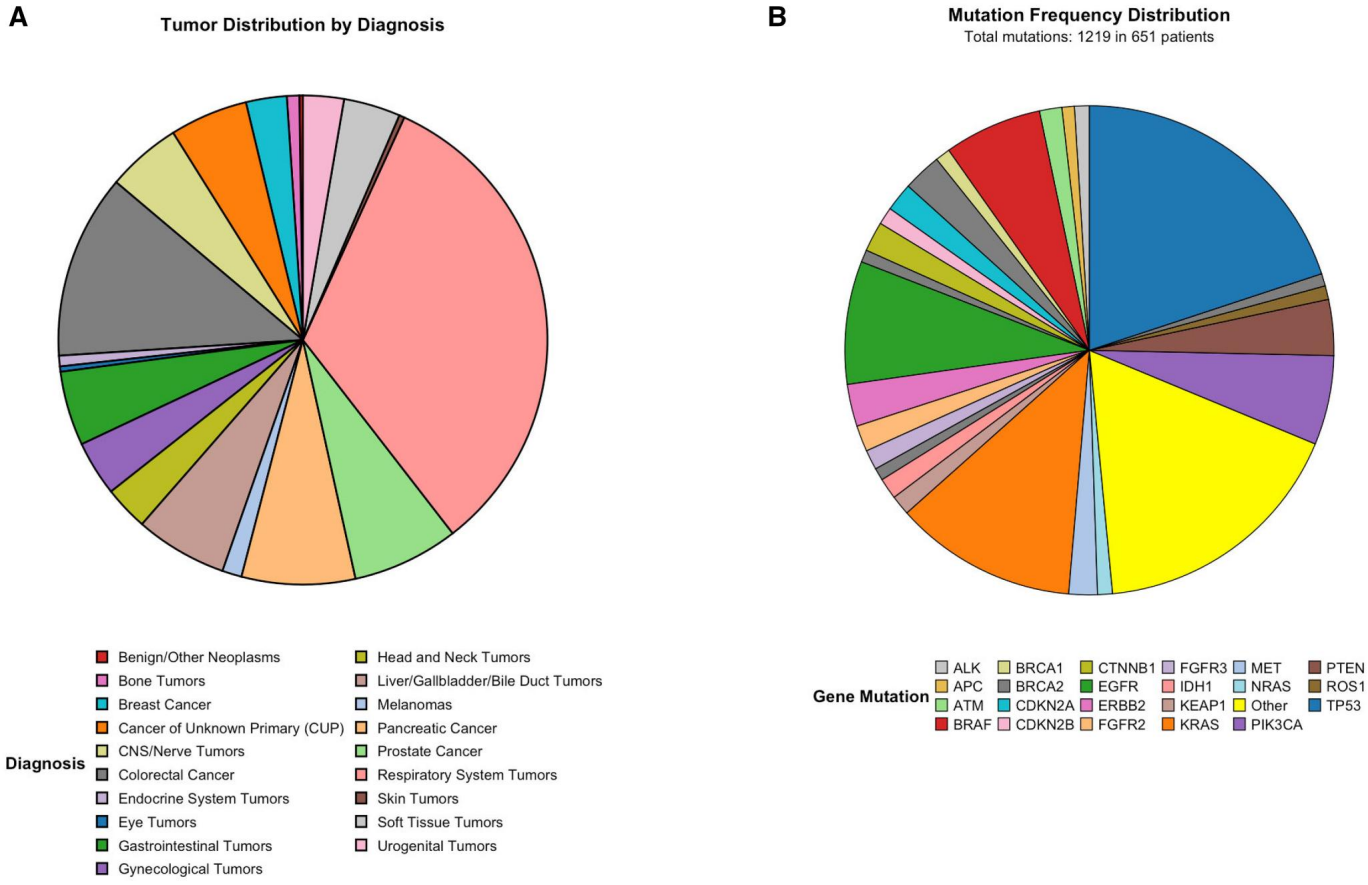
(A) Distribution of tumor entities among patients referred to the Molecular Tumor Board. This pie chart illustrates the distribution of tumor diagnoses among patients discussed at the Molecular Tumor Board meeting. The largest proportion was respiratory system tumors (light red), followed by colorectal cancer (grey), pancreatic cancer (light orange), and prostate cancer (light green). A diverse range of other malignancies, including breast cancer, gynecological tumors, head and neck tumors, and CUP (cancer of unknown primary), were also represented. This distribution highlights the broad range of diagnoses covered by precision oncology approaches and the importance of molecular diagnostics in treating different types of cancer. (B) Gene mutation frequency distribution among molecularly characterized patients This pie chart illustrates the frequency with which gene mutations were detected across a molecularly profiled oncology cohort comprising 651 patients and a total of 1219 mutations. The most prevalent alterations were observed in TP53 (blue), KRAS (orange), EGFR (green), and BRAF (red), reflecting common driver mutations across various tumor types. Additional recurrent mutations include PIK3CA, PTEN, ERBB2, and BRCA2, among others. Mutations occurring below the APC frequency threshold (frequency = 10) are categorized as “Other,” emphasizing the molecular heterogeneity of the cohort.

From January 2016 to December 2022, a total of 1074 MTB case discussions covering 949 unique individuals were presented to the UCC Hamburg MTB. Of these, 854/949 (90%) had a solid tumor (Table 1) and formed the main analysis cohort. Annual MTB referrals increased almost 10-fold from 17 in 2016 to approximately 233 in 2022. Overall, 54% of individuals were male and 46% female; median age at MTB was 59 years (IQR 48-68). The most common tumor entities were respiratory tract tumors (33%), colorectal cancer (12%), pancreatic cancer (8%), and prostate cancer (7%) (Figure 1A, Table S1). The median interval from first diagnosis to MTB discussion was 404 days (IQR 81-915), and patients were heavily pretreated with a median of 2 prior systemic therapy lines (mean 2.6; range 0-18). ECOG performance status ≥2 was documented in 24% at MTB presentation (Table 1).

**Table 1.** Patient characteristics by therapy recommendation category (1st priority targeted therapy, 2nd priority targeted therapy, non-targeted therapy, no therapy).

Variable	*N*	1st priority targeted therapy, *N* = 205	2nd priority targeted therapy, *N* = 59	Therapy, non-targeted, *N* = 62	No therapy, *N* = 528	Overall, *N* = 854	***P*-value** ^a^	***q*-value** ^b^
**Age at MTB**	854						.10	0.2
** Median [IQR]**		58.0 [49.0, 69.0]	59.0 [48.5, 68.5]	63.0 [52.3, 71.8]	58.0 [46.0, 67.0]	59.0 [48.0, 68.0]		
**Sex, n (%)**	854						.3	0.3
** M**		107 (52)	28 (47)	40 (65)	283 (54)	458 (54)		
** F**		98 (48)	31 (53)	22 (35)	245 (46)	396 (46)		
**ECOG performance status, *n* (%)**	540						**.013**	0.026
** ≥2**		43 (29)	8 (19)	16 (38)	60 (19)	127 (24)		
** 0-1**		105 (71)	34 (81)	26 (62)	248 (81)	413 (76)		
** Unknown**		57	17	20	220	314		
**Year of MTB, *n* (%)**	854						**<.001**	<0.001
** 2016-2018**		64 (31)	13 (22)	29 (47)	47 (9)	153 (18)		
** 2019-2020**		78 (38)	17 (29)	27 (44)	142 (27)	264 (31)		
** 2021-2022**		63 (31)	29 (49)	6 (10)	339 (64)	437 (51)		
**Level of evidence, *n* (%)**	228						**.002**	0.006
** m1A**		96 (63)	13 (33)	28 (78)	0 (NA)	137 (60)		
** m1B**		15 (10)	5 (13)	1 (3)	0 (NA)	21 (9)		
** m1C**		11 (7)	3 (8)	3 (8)	0 (NA)	17 (7)		
** ≥m2**		31 (20)	18 (46)	4 (11)	0 (NA)	53 (23)		
** Unknown**		52	20	26	528	626		
**Tissue origin, *n* (%)**	735						.4	0.5
** Liquid biopsy**		4 (2)	1 (2)	0 (0)	10 (2)	15 (2)		
** Local recurrence**		9 (5)	4 (7)	5 (8)	23 (5)	41 (6)		
** Metastasis**		71 (36)	23 (40)	26 (43)	190 (45)	310 (42)		
** Primary tumor**		114 (58)	30 (52)	29 (48)	196 (47)	369 (50)		
** Unknown**		7	1	2	109	119		
**MSI Testing, *n* (%)**	854						**<.001**	<0.001
** Done**		164 (80)	56 (95)	52 (84)	483 (91)	755 (88)		
** Not done**		41 (20)	3 (5)	10 (16)	45 (9)	99 (12)		
**PD-L1 tumor cells, *n* (%)**	854						**.006**	0.015
** Done**		116 (57)	34 (58)	36 (58)	236 (45)	422 (49)		
** Not done**		89 (43)	25 (42)	26 (42)	292 (55)	432 (51)		
**Lines of therapy before MTB**	853						.3	0.4
** Median [IQR]**		2.0 [1.0, 4.0]	1.0 [0.0, 4.5]	2.0 [1.0, 4.0]	2.0 [0.0, 4.0]	2.0 [0.0, 4.0]		
** Unknown**		0	0	0	1	1		
**Timing of MTB (d)**	847						**<.001**	<0.001
** Median [IQR]**		340.0 [44.0, 897.0]	318.0 [58.5, 712.0]	185.0 [41.0, 529.0]	463.0 [125.0, 1,006.0]	404.0 [81.0, 915.0]		
** Unknown**		0	0	0	7	7		
**Response duration vs PFS_prior_, *n* (%)**	182						.6	0.6
** ≤1.3**		54 (39)	13 (31)	1 (50)	0 (NA)	68 (37)		
** >1.3**		84 (61)	29 (69)	1 (50)	0 (NA)	114 (63)		
** Unknown**		67	17	60	528	672		
**Best response, *n* (%)**	274						**.017**	0.029
** Complete remission (CR)**		6 (3)	0 (0)	4 (8)	0 (NA)	10 (4)		
** Death**		23 (13)	6 (14)	6 (13)	0 (NA)	35 (13)		
** Partial remission (PR)**		50 (27)	5 (11)	14 (29)	0 (NA)	69 (25)		
** Progressive disease (PD)**		48 (26)	22 (50)	17 (35)	0 (NA)	87 (32)		
** Stable disease (SD)**		55 (30)	11 (25)	7 (15)	0 (NA)	73 (27)		
** Unknown**		23	15	14	528	580		

a Kruskal–Wallis rank sum test; Pearson’s Chi-squared test.

b Benjamini & Hochberg correction for multiple testing.

*P*-values below 0.05 are printed bold.

### Molecular diagnostics

In the solid tumor cohort (*n* = 854), 90.75% of MTB recommendations were informed by tumor sequencing diagnostics. Molecular testing was predominantly tissue-based (primary tumor 50%, metastasis 42%, local recurrence 6%); liquid profiling contributed in approximately 2% of cases. The most frequently used assay was a customized multigene routine panel including FISH-based screening for amplifications and gene fusions (44%), while broader panels were increasingly adopted from 2019 onward.

### Molecular alterations and treatment recommendations

Across 651/854 molecularly characterized individuals, 1219 mutations in 143 genes were reported. The most frequently altered genes were TP53 (*n* = 243), KRAS (*n* = 147), EGFR (*n* = 99), BRAF (*n* = 79), PIK3CA (*n* = 72), and PTEN (*n* = 45) (Figure 1B, Table S2). In addition, 50 gene fusions/translocations across 18 genes were identified, most commonly involving ROS1 (*n* = 9), ALK (*n* = 8), RET (*n* = 7), and NRG1 (*n* = 9). Furthermore, 98 copy number variations in 36 genes were detected; MET was most frequently altered (*n* = 23), followed by HER2 (*n* = 22) and FGFR1 (*n* = 8). MSI testing was performed in 88% of cases and PD-L1 tumor cell immunohistochemistry in 49%.

### Actionability and evidence levels

Overall, molecular findings supported 623 MTB treatment recommendations. Of these, 231/623 (37%) were issued in the absence of an actionable/targetable alteration. Across targeted recommendations, the most common matches involved EGFR alterations (eg, osimertinib, afatinib) and PIK3CA or BRAF alterations (eg, alpelisib, dabrafenib). Each targeted treatment recommendation was assigned an evidence level according to the published framework.^25^ In the majority of recommendations, the evidence level was m1A (60%).

### Prioritization of MTB recommendations

When multiple molecularly matched options were available, MTB prioritization did not rely on evidence level alone. Priority 1 was assigned to the option judged most appropriate after multidisciplinary discussion considering (i) strength and relevance of evidence level, (ii) biological plausibility/on-target relevance for the tumor type and alteration, (iii) regulatory status and practical accessibility (including reimbursement feasibility), (iv) anticipated toxicity in the individual clinical context and comorbidities, and (v) feasibility (eg, organ function requirements, drug-drug interactions) and availability of clinical trials. Priority 2 represented the next best alternative when the top-ranked option could not be pursued or was expected to be less suitable based on these criteria. This approach also applied when evidence levels were comparable (eg, multiple off-label options with similar evidence grades).

### Follow-up and implementation

At the time of analysis, structured follow-up allowing assessment of post-MTB therapy and outcomes was available for 326/854 (38%) individuals (Table 1). Within this follow-up cohort, priority 1 targeted therapy was implemented in 205/326 (62%) and priority 2 targeted therapy in 59/326 (18%). Non-targeted systemic therapy was implemented in 62/326 (19%). Relative to the entire solid tumor cohort, this corresponds to 205/854 (24%) priority 1 and 59/854 (7%) priority 2 targeted therapy implemented; overall, targeted therapy implementation was 264/854 (31%). Among all targeted therapy recipients (*n* = 264), 205 (78%) received a priority 1 match and 59 (22%) a priority 2 match. In most cases, targeted therapy was administered “off-label” (51%), while 4% received treatment within a clinical trial.

### Clarification regarding “off-label” versus evidence tiers

In this cohort, “off-label” refers to the regulatory status of the prescribed drug in Germany (ie, outside the approved label for the given tumor entity/indication) and is not synonymous with a specific evidence tier. Evidence levels for MTB recommendations were assigned independently and according to the applied framework.^25^ Therefore, “off-label in Germany” cannot be mapped 1:1 to US tier labels (such as Tier 1C) without explicitly specifying the US system used and performing a formal crosswalk, which was not part of this analysis.

### Response outcomes

Among patients with documented response assessments, priority 1 targeted therapy was associated with an objective response rate (ORR) of 30.8% (partial response 27.5%, complete response 3.3%) and a disease control rate (DCR) of 61%. Priority 2 targeted therapy showed a lower ORR of 11.4% (no complete responses) and a DCR of 36.4%. Non-targeted therapies showed an ORR of 37.5% (complete response 8.3%) and a DCR of 52.1% (*P* = .017, chi-square test).

### Conventional time-to-event analyses from MTB date (t_0_)

**Figure 2.**
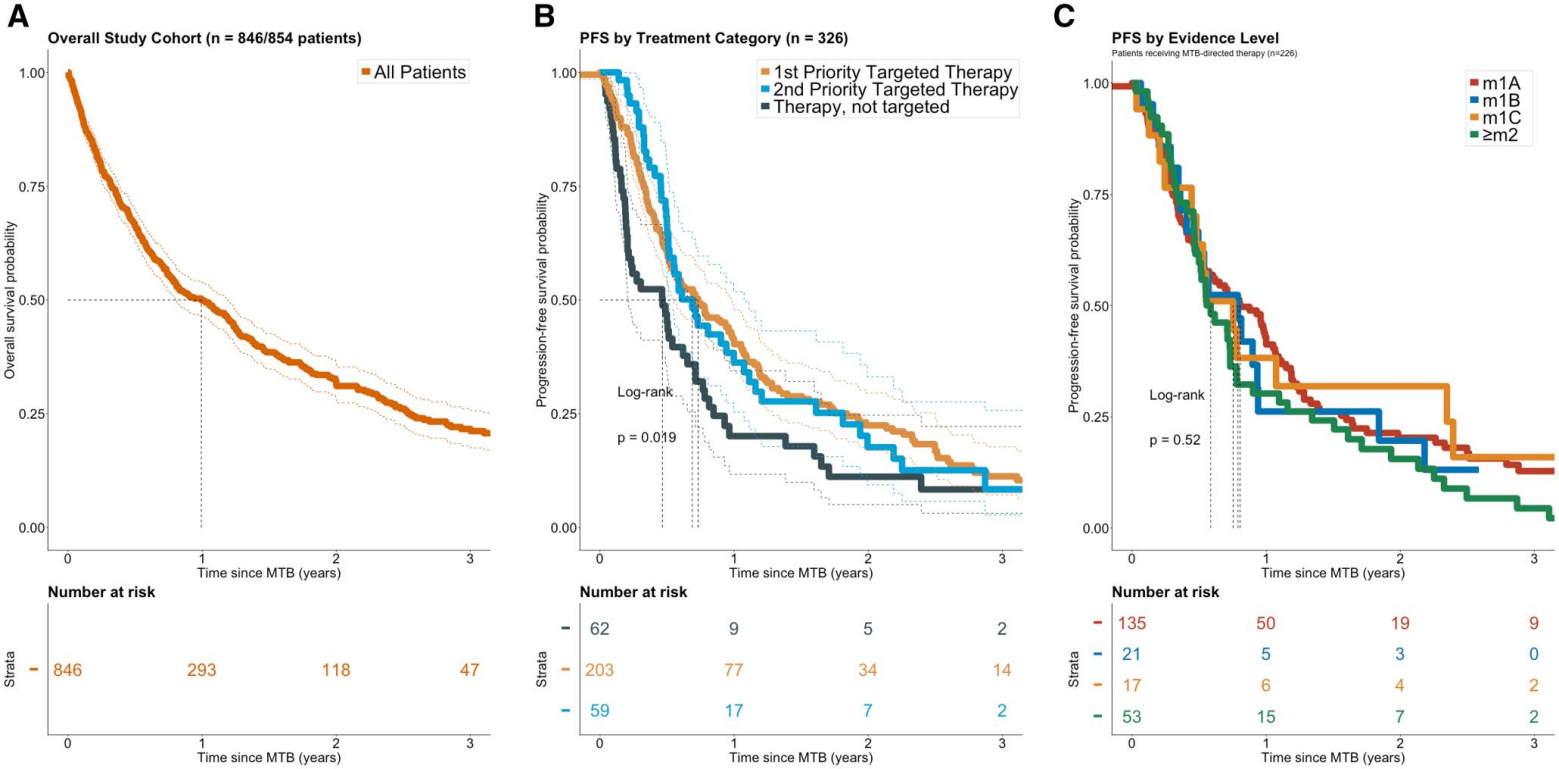
(A) Overall survival (OS_MTB_) of entire study cohort (*n* = 846) from the date of the molecular tumor board (MTB) presentation (t_0_). The shaded areas represent 95% confidence intervals. (B) Progression-free survival (PFS_MTB_) from the MTB date in patients with available follow-up data, categorized by treatment (*n* = 346). The median PFS_MTB_ differed significantly between the groups (log-rank *P* = .019), with the not-targeted therapy group showing the shortest PFS_MTB_. (C) PFS_MTB_ in patients receiving MTB-directed therapy, stratified by evidence level (*n* = 226; m1A, m1B, m1C, and ≥ m2). No significant differences were observed between evidence level categories (log-rank *P* = .52).

In 326 individuals with available follow-up, 1-, 2-, and 3-year overall survival (OS_MTB_) were 50.0% (95% CI, 46.4-53.8), 31.6% (95% CI, 27.9-35.8) and 21.6% (95% CI, 18.0-26.1), respectively (Figure 2A). Progression-free survival from MTB (PFS_MTB_) differed between therapy strata. Median PFS_MTB_ for priority 1 targeted therapy was 267 days (8.9 months; *n* = 204; Figure 2B). In univariate analysis, both priority 1 targeted therapy (HR 0.64, 95% CI, 0.47-0.88, *P* = .006) and priority 2 targeted therapy (HR 0.67, 95% CI, 0.45-1.00, *P* = .050) were associated with improved PFS_MTB_ compared with non-targeted systemic therapy.

### Within-person benefit (PFS-ratio)

For within-person benefit assessment, progression-free survival on the implemented MTB-directed therapy was compared with progression-free survival on the immediately preceding systemic therapy line (PFS_prior_) using the PFS-Ratio (PFS_MTB_/PFS_prior_). A PFS-ratio >1.3 was observed in 61% of targeted therapy recipients. Among the 47 individuals who received targeted treatment off-label and had both components available, the ratio was >1.3 in 62%.

### Landmark treatment setting and evidence level

When calculated from therapy start in those with available data (PFS_targ_), median PFS_targ_ was 210 days (7.0 months) in the off-label setting and 312 days (10.4 months) in the trial setting (*n* = 79). The impact of evidence level on PFS_MTB_ was examined (Figure 2C). No clear advantage was observed with increasing evidence level (HR vs m1A: m1B HR = 1.11 [95% CI, 0.66-1.86], *P* = .69; m1C HR = 0.91 [95% CI, 0.51-1.62], *P* = .74; ≥m2 HR = 1.27 [95% CI, 0.90-1.78], *P* = .17).

### Further survival subsets and conventional comparisons

**Figure 3.**
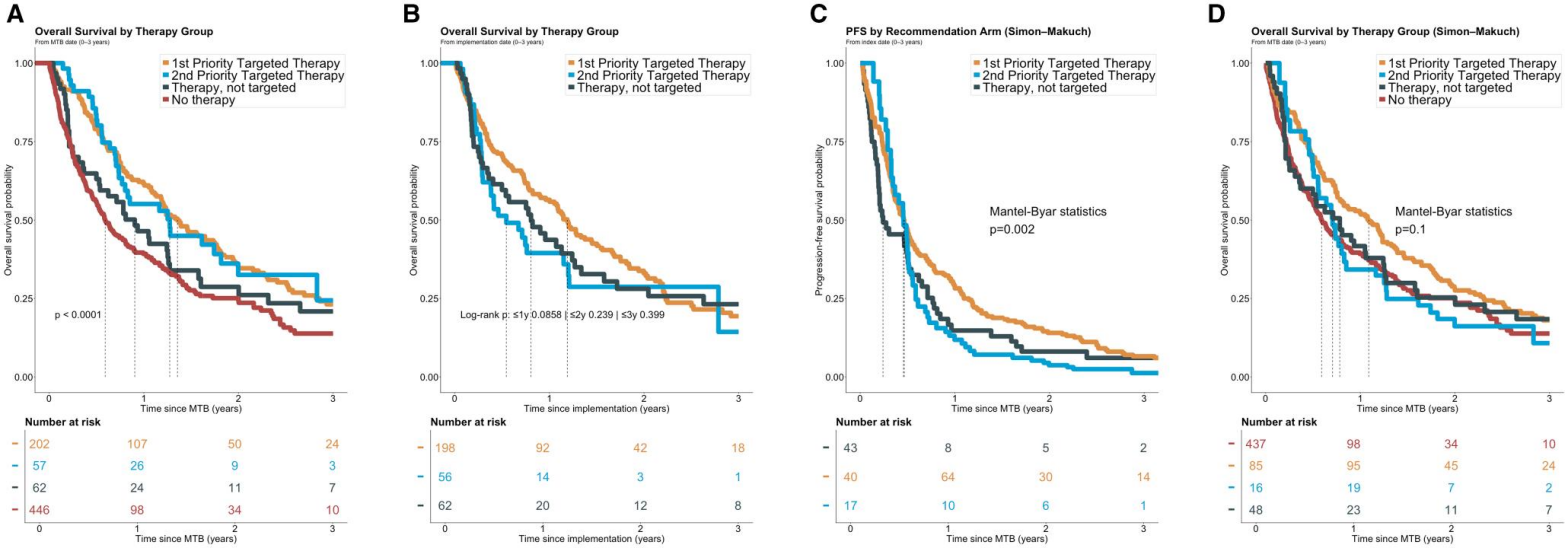
(A) OS_MTB_ from the date of the molecular tumor board (MTB—t_0_) presentation for patients in the first and second recommended therapy and the not targeted treated and no therapy groups (*n* = 846; median follow-up: 18.8 months; median overall survival (OS_MTB_): 495, 465, 330, and 216 days, respectively). (B) OS_targ_ from the date of therapy implementation for patients in the different groups (*n* = 326; median follow-up: 28.5 months; median OS: 434, 199, and 294 days respectively). (C) PFS_SimonMakuch_ estimated by Mantel–Byar and depicted in Simon–Makuch plots from the index date with treatment initiation as a time-dependent covariate according to the recommended treatment (Mantel–Byar *P* = .002). (D) OS_SimonMakuch_ estimated by Mantel–Byar and depicted in Simon–Makuch plots from the MTB date according to the therapy group (Mantel–Byar *P* = .10) with treatment initiation as a time-dependent covariate.

When calculated from the MTB date in the entire solid tumor cohort (*n* = 854), median OS_MTB_ was 495 days (16.5 months) for priority 1 targeted therapy, 465 days (15.5 months) for priority 2 targeted therapy, 330 days (11.0 months) for non-targeted therapy, and 216 days (7.2 months) for individuals without post-MTB systemic therapy (log-rank *P* < .0001; Figure 3A). In a Cox regression using the priority 1 group as reference, no significant difference in survival was observed for the priority 2 group (HR 0.98, 95% CI, 0.67-1.43, *P* = .90), whereas individuals without a recommendation suggesting targeted therapy showed a non-significant trend towards worse survival (HR 1.35, 95% CI, 0.95-1.91, *P* = .09). Individuals without any post-MTB systemic therapy documented experienced significantly shorter survival (HR 1.73, 95% CI, 1.40-2.15, *P* < .001). When calculated from the therapy start date among those with documented implementation (*n* = 326), median overall survival was 434 days (14.4 months) for priority 1 targeted therapy, 199 days (6.6 months) for priority 2 targeted therapy, and 294 days (9.8 months) for non-targeted therapy; differences were not statistically significant for priority 1 versus priority 2 (HR 1.38, 95% CI 0.94-2.02, *P* = .098) or for priority 1 versus non-targeted therapy (HR 1.15, 95% CI, 0.81-1.64, *P* = .43) (Figure 3B).

### Time-dependent modeling to address immortal time bias

Because therapy initiation occurs after MTB discussion, conventional Kaplan–Meier estimates grouped by post-baseline therapy are prone to immortal person-time bias. Mantel-Byar analyses with therapy initiation modeled as a time-dependent covariate mitigate this bias and were feasible in our dataset. In Mantel-Byar time-dependent analyses depicted as Simon–Makuch plots, progression-free survival separation favored the priority 1 group over both priority 2 and non-targeted therapy (Mantel-Byar *P* = .002; Figure 3C). At 1 year, PFS_SimonMakuch_ was 28% for priority 1, 12% for priority 2, and 15% for non-targeted therapy. In the corresponding time-dependent Cox model (*n* = 322), priority 1 showed a trend towards reduced risk of progression or death compared with non-targeted therapy (HR 0.76, 95% CI, 0.55-1.04, *P* = .088), while priority 2 did not differ significantly (HR 1.31, 95% CI, 0.88-1.96, *P* = .18). For overall survival, Mantel-Byar comparisons did not reach statistical significance across groups (*P* = .1; Figure 3D). At 1 year, OS_SimonMakuch_ was 52% in the priority 1 group, 39% in the no therapy group, 42% in the non-targeted group, and 34% in the priority 2 group. In the time-dependent Cox model (*n* = 752), priority 1 targeted therapy was associated with a significantly lower risk of death compared with no post-MTB systemic therapy documented (HR 0.80, 95% CI, 0.64-0.99, *P* = .038), whereas priority 2 (HR 1.16, *P* = .43) and non-targeted therapy (HR 0.92, *P* = .62) were not significantly different.

## Discussion

In predictive oncology, molecular diagnostics, including massively parallel tumor sequencing, continues to reshape cancer classification and treatment selection. Real-world outcomes analyses of MTBs have been reported for nearly a decade, but most cohorts are single-center or bicenter and show substantial heterogeneity in implementation rates and clinical outcomes.^29^^,^^35–37^ A recent meta-analysis summarizing 34 studies with more than 10 000 individuals treated between 2020 and 2024 reported a pooled implementation rate of 20.8%, a median overall survival (OS) after therapy implementation of 13.5 months, and a median progression-free survival (PFS) of 4.5 months, with objective response rates (ORR) ranging from 5%-57%.^30^ In our cohort, the implementation rate of the MTB top-priority recommendation was 24% (205/854), and overall targeted therapy implementation was 31% (264/854), placing our program within the published range. Outcome measures were directionally favorable for selected endpoints, including a median OS of 15.5-16.5 months for individuals receiving targeted therapies, a median PFS of 9 months for priority 1, an ORR up to 30.8%, and a disease control rate (DCR) up to 61% for those treated according to the MTB top-priority recommendation (with lower ORR/DCR of 11.4%/36.4% for those receiving an alternative targeted therapy).

A central methodological challenge in MTB outcome research is immortal person-time bias. MTB-guided therapies typically start after MTB discussion; therefore, if exposure groups are defined by a post-baseline event (therapy initiation), conventional Kaplan–Meier comparisons can overestimate treatment benefit. In our dataset, therapy start occurred after the MTB index date (t_0_), and as expected, Kaplan–Meier estimates yielded higher apparent survival probabilities than time-dependent Mantel-Byar estimates, demonstrating both the magnitude and direction of bias (Table 2). Mantel-Byar analysis addresses this problem by treating therapy initiation as a time-dependent exposure and allocating person-time prior to therapy start to the unexposed risk set.^33^^,^^34^ Simon–Makuch plots provide the corresponding visualization of these modified Kaplan–Meier curves under time-dependent exposure, transparently showing re-allocation of individuals across risk sets at therapy start.^33^^,^^34^

**Table 2.** Comparison of time to event estimation following time-dependent covariation of implementation of MTB-directed therapy versus Kaplan–Meier-estimation. T_0_ was defined in both case in consultation with the molecular tumor board; in the Mantel–Byar approach, however, treatment initiation is accounted for only from the later (post-treatment) start time.

Group	Year 1	Year 2	Year 3
**PFS Mantel–Byar (PFS_SimonMakuch_)**			
** Priority 1**	28.2 [22.4-35.6]	14.0 [9.9-19.8]	6.6 [3.9-11.1]
** Priority 2**	11.8 [5.6-24.9]	3.7 [1.2-11.2]	1.2 [0.2-8.0]
** Non-targeted alternative**	14.8 [7.9-27.8]	8.1 [3.4-19.3]	6.1 [2.2-17.1]
**PFS Kaplan–Meier (PFS_MTB_)**			
** Priority 1**	40.3 [33.9-47.8]	22.5 [17.1-29.5]	11.2 [7.1-17.7]
** Priority 2**	36.2 [25.4-51.8]	17.6 [9.4-33.1]	8.4 [2.7-25.8]
** Non-targeted alternative**	20.1 [11.7-34.4]	11.2 [5.0-24.7]	8.4 [3.2-22.2]

After correction for immortal person-time, we observed a significant PFS signal favoring implementation of the MTB top-priority targeted recommendation compared with both alternative (priority 2) targeted therapy and non-targeted systemic therapy (Mantel-Byar *P* = .002). One-year PFS was 28% for priority 1 targeted therapy, 12% for priority 2, and 15% for non-targeted therapy. Overall survival differences were directionally consistent, with one-year OS of 52% for priority 1 compared with 42% for non-targeted therapy and 34% for priority 2, but did not reach statistical significance in the Mantel-Byar comparison (*P* = .1). In a time-dependent Cox model, the priority 1 group was associated with a lower risk of death versus no post-MTB systemic therapy (HR 0.80, 95% CI, 0.64-0.99, *P* = .038), whereas priority 2 and non-targeted treatment were not significantly different.

Our findings also align with pragmatic within-person benefit assessments used in heterogeneous MTB cohorts. The progression-free survival ratio (PFS-ratio) has been proposed as a metric to benchmark whether an MTB-guided therapy exceeds the benefit of the immediately preceding therapy line within the same individual.^26^ In the meta-analysis, a pooled PFS2/PFS1 ratio ≥ 1.3 (14 reports) was observed in 38% (33%-44%).^30^ Using the analogous within-person definition in our cohort (PFSMTB/PFSprior), a ratio > 1.3 was observed in 63% of targeted therapy recipients, providing a complementary signal of clinical activity in a setting where randomized comparators are rarely feasible.

Important limitations and residual biases remain. Treatment allocation was not randomized and depends on clinical feasibility, access and reimbursement, evolving standards of care over the study period, and patient and physician preferences. Individuals selected for targeted therapy tended to have more favorable baseline characteristics, including better performance status, while ECOG status was incompletely documented; additional unmeasured confounders likely include comorbidity burden, organ function, and tumor kinetics. Therefore, the observed associations should be interpreted as real-world signals rather than proof of efficacy.

Finally, we consider remaining room for multivariable modeling. In principle, time-dependent Cox models could be adjusted for baseline covariates such as age, sex, tumor entity, year of MTB, ECOG status, and number of prior therapy lines; approaches such as inverse probability weighting or marginal structural models could be considered when time-varying confounding is suspected. In the present analysis, multivariable adjustment was limited by missingness in ECOG and other baseline variables and by potential collinearity between clinical status, tumor type, and access to targeted drugs. Prospective harmonization of baseline covariates and a prespecified missing-data strategy in MTB registries would improve the robustness of adjusted estimates in future work.

Off-label use was frequent and reflects the European regulatory context and the pragmatic aim of MTBs to expand access to matched therapies beyond approvals. Regulatory status (on-label vs off-label) is not synonymous with evidence level. Evidence tiers and annotation frameworks differ between jurisdictions and are not directly interchangeable; therefore, off-label status in Germany should not be mapped to US tier labels without explicitly specifying the US system and performing a formal crosswalk. We therefore report evidence levels as applied by the MTB using the selected framework^25^ and avoid jurisdiction-specific tier translations.

Taken together, this real-world analysis of the UCC Hamburg MTB (2016-2022) provides implementation patterns and outcome measures in a heterogeneous, heavily pretreated cohort and demonstrates that time-dependent methods might be used for retrospective MTB outcome analyses whenever therapy initiation occurs after the time origin. Centering Mantel-Byar comparisons with Simon–Makuch visualization offers a transparent approach to mitigate immortal person-time bias and to contextualize treatment-associated outcome differences, while highlighting remaining confounding and data-completeness challenges that should be addressed in future, more standardized evaluations.

## Supplementary Material

oyag078_Supplementary_Data

## Data Availability

The data underlying this article cannot be shared publicly due to the privacy of individuals that participated in the study. The data will be shared on reasonable request with the corresponding author.
