# Supplementary material for: Time dependent outcomes modeling in a real-world analysis of the molecular tumor board at University Cancer Center Hamburg (2016-2022)
Source: Oncologist. 2026 Mar 31;31(5):oyag078. doi: 10.1093/oncolo/oyag078 (PMC13070728; doi:10.1093/oncolo/oyag078)
Supplement: oyag078_Supplementary_Data [file oyag078_supplementary_data.docx]

| Diagnosis | Count | Diagnosis | Count |
| --- | --- | --- | --- |
| Benign/Other Neoplasms | 2 | Bone Tumors | 7 |
| Breast Cancer | 23 | Cancer of Unknown Primary (CUP) | 44 |
| CNS/Nerve Tumors | 42 | Colorectal Cancer | 104 |
| Endocrine System Tumors | 6 | Eye Tumors | 3 |
| Gastrointestinal Tumors | 42 | Gynecological Tumors | 31 |
| Head and Neck Tumors | 25 | Liver/Gallbladder/Bile Duct Tumors | 52 |
| Melanomas | 11 | Pancreatic Cancer | 64 |
| Prostate Cancer | 60 | Respiratory System Tumors | 279 |
| Skin Tumors | 3 | Soft Tissue Tumors | 32 |
| Urogenital Tumors | 23 | Not specified | 1 |

Table S1: Number of diagnoses among all patients (n = 854)

| Gene | **n** | **Gene** | **n** |
| --- | --- | --- | --- |
| TP53 | 243 | CBL | 1 |
| KRAS | 147 | CCND3 | 1 |
| EGFR | 99 | CD274 | 1 |
| BRAF | 79 | CDH1 | 1 |
| PIK3CA | 72 | CDK12 | 1 |
| PTEN | 45 | CHD2 | 1 |
| ERBB2 | 34 | CSF1R | 1 |
| BRCA2 | 31 | CTNNA1 | 1 |
| CTNNB1 | 24 | DCUN1D1 | 1 |
| CDKN2A | 23 | DDX41 | 1 |
| MET | 23 | DNMT3A | 1 |
| FGFR2 | 21 | EP300 | 1 |
| ATM | 18 | ERBB4 | 1 |
| IDH1 | 17 | ERCC2 | 1 |
| FGFR3 | 16 | ERCC4 | 1 |
| KEAP1 | 16 | FANCD2 | 1 |
| CDKN2B | 14 | FAS | 1 |
| ALK | 12 | FBXW7 | 1 |
| BRCA1 | 12 | FH | 1 |
| NRAS | 12 | GATA3 | 1 |
| ROS1 | 11 | GRIN2A | 1 |
| APC | 10 | GRM3 | 1 |
| DDR2 | 10 | H3F3A | 1 |
| FGFR4 | 10 | HIST1H3B | 1 |
| STK11 | 10 | IKBKE | 1 |
| FGFR1 | 9 | INPP4B | 1 |
| AR | 7 | KDM6A | 1 |
| MAP2K1 | 7 | KDR | 1 |
| RB1 | 7 | KEL | 1 |
| IDH2 | 6 | KMT2B | 1 |
| RET | 6 | MAP2K4 | 1 |
| PALB2 | 5 | MAPK1 | 1 |
| PDGFRA | 5 | MED12 | 1 |
| ATR | 4 | MLH1 | 1 |
| CHEK2 | 4 | MSH6 | 1 |
| KIT | 4 | MST1 | 1 |
| NTRK1 | 4 | NBN | 1 |
| ATRX | 3 | NCOR1 | 1 |
| HRAS | 3 | NFE2L2 | 1 |
| JAK1 | 3 | NOTCH2 | 1 |
| JAK3 | 3 | NOTCH3 | 1 |
| MTOR | 3 | NRG1 | 1 |
| NF1 | 3 | NSD1 | 1 |
| NF2 | 3 | NTRK3 | 1 |
| NTRK2 | 3 | PBRM1 | 1 |
| ASXL1 | 2 | PDGFRB | 1 |
| AURKA | 2 | PIK3C2G | 1 |
| BRIP1 | 2 | PIK3C3 | 1 |
| CDKN1B | 2 | PIK3R1 | 1 |
| CIC | 2 | PLK2 | 1 |
| CREBBP | 2 | POLD1 | 1 |
| FANCA | 2 | POLE | 1 |
| FLT1 | 2 | PPARG | 1 |
| FOXA1 | 2 | PREX2 | 1 |
| GATA4 | 2 | PTCH1 | 1 |
| GNAS | 2 | PTPN11 | 1 |
| KMT2D | 2 | RAD50 | 1 |
| MAP3K13 | 2 | RAD51 | 1 |
| MSH2 | 2 | RAD51C | 1 |
| NOTCH1 | 2 | RAD51D | 1 |
| PMS2 | 2 | RBM10 | 1 |
| SETD2 | 2 | SMARCB1 | 1 |
| SF3B1 | 2 | SOX9 | 1 |
| SMAD4 | 2 | STAG2 | 1 |
| TSC2 | 2 | STAT3 | 1 |
| ACVR1B | 1 | TERT | 1 |
| ARAF | 1 | TGFBR2 | 1 |
| BAP1 | 1 | TMPRSS2 | 1 |
| BCOR | 1 | TSC1 | 1 |
| BIRC3 | 1 | U2AF1 | 1 |
| BRD4 | 1 | VHL | 1 |
| CARD11 | 1 |  |  |

Table S2: Mutations among all 1219 mutations.
